# Decreased circulating CTRP3 levels in acute and chronic cardiovascular patients

**DOI:** 10.1007/s00109-024-02426-8

**Published:** 2024-03-04

**Authors:** Andreas Schmid, Sabine Pankuweit, Ann-Kathrin Vlacil, Sören Koch, Benedikt Berge, Praveen Gajawada, Manfred Richter, Kerstin Troidl, Bernhard Schieffer, Andreas Schäffler, Karsten Grote

**Affiliations:** 1 Department of Internal Medicine III, Giessen University Hospital, Giessen, Germany; 2 Cardiology and Angiology, Philipps-University Marburg, Marburg, Germany; 3 Department of Cardiac Surgery, Kerckhoff Heart Center, Bad Nauheim, Germany; 4 Department of Life Sciences and Engineering, TH Bingen, University of Applied Sciences, Bingen Am Rhein, Germany; 5 Department of Vascular and Endovascular Surgery, Cardiovascular Surgery Clinic, University Hospital Frankfurt and Wolfgang Goethe University Frankfurt, Frankfurt, Germany

## Abstract

**Abstract:**

C1q/TNF-related protein 3 (CTRP3) represents an adipokine with various metabolic and immune-regulatory functions. While circulating CTRP3 has been proposed as a potential biomarker for cardiovascular disease (CVD), current data on CTRP3 regarding coronary artery disease (CAD) remains partially contradictory. This study aimed to investigate CTRP3 levels in chronic and acute settings such as chronic coronary syndrome (CCS) and acute coronary syndrome (ACS). A total of 206 patients were classified into three groups: CCS (n = 64), ACS having a first acute event (ACS-1, n = 75), and ACS having a recurrent acute event (ACS-2, n = 67). The control group consisted of 49 healthy individuals. ELISA measurement in peripheral blood revealed decreased CTRP3 levels in all patient groups (p < 0.001) without significant differences between the groups. This effect was exclusively observed in male patients. Females generally exhibited significantly higher CTRP3 plasma levels than males. ROC curve analysis in male patients revealed a valuable predictive potency of plasma CTRP3 in order to identify CAD patients, with a proposed cut-off value of 51.25 ng/mL. The sensitivity and specificity of prediction by CTRP3 were congruent for the subgroups of CCS, ACS-1, and ACS-2 patients. Regulation of circulating CTRP3 levels in murine models of cardiovascular pathophysiology was found to be partly opposite to the clinical findings, with male mice exhibiting higher circulating CTRP3 levels than females. We conclude that circulating CTRP3 levels are decreased in both male CCS and ACS patients. Therefore, CTRP3 might be useful as a biomarker for CAD but not for distinguishing an acute from a chronic setting.

**Key messages:**

- CTRP3 levels were found to be decreased in both male CCS and ACS patients compared to healthy controls. Plasma CTRP3 has a valuable predictive potency in order to identify CAD patients among men and is therefore proposed as a biomarker for CAD but not for distinguishing between acute and chronic settings.
- Regulation of circulating CTRP3 levels in murine models of cardiovascular pathophysiology was found to be partly opposite to the clinical findings in men.

**Supplementary Information:**

The online version contains supplementary material available at 10.1007/s00109-024-02426-8.

## Introduction

Cardiovascular diseases (CVD) are a major health issue and among the most common causes of mortality [1] with a strong association with obesity and metabolic syndrome [2, 3]. Elevated body-mass-index (BMI) represents a significant risk factor for severe CVD and mortality, although the correlation between obesity and fatal outcomes in CVD is complex and affected by the presence of further comorbidities [2]. Among CVD, coronary artery disease (CAD) emerging from endothelial-derived inflammation and subsequent atherosclerosis, fueled by chronically elevated levels of low-density lipoprotein cholesterol (LDL) [4], represents a widespread and severe health issue [5]. Acute events of myocardial infarction (MI) – but also stroke – are mostly a consequence of atherosclerosis, with plaque rupture and superficial erosion typically causing localized, often occlusive thrombus formation.

In the context of metabolic syndrome, adipose tissue plays a fundamental role. It represents a potent endocrine organ that is involved in the regulation of essential physiological processes comprising both metabolic and immunological mechanisms [6–8]. These regulatory effects are mediated by the secretion of soluble proteins – the so-called adipokines – exerting endocrine, paracrine, and autocrine activity [9]. Importantly, anti- and pro-inflammatory adipokines play a major role in CVD, linking these morbidities with obesity [10]. C1q/TNF-related protein 3 (CTRP3) is a pleiotropic adipokine involved in multiple and diverse physiological processes [11]. In addition to its beneficial properties concerning the regulation of glucose and lipid metabolism [12, 13] as well as inflammation [14, 15], inverse associations of circulating CTRP3 with cardio-metabolic risk factors and protective effects in cardiovascular diseases have been reported previously [11, 16], including atherosclerosis and acute coronary syndrome (ACS) [17, 18]. CTRP3 attenuates high-pressure-induced cardiac hypertrophy [19] as well as high-glucose-induced endothelial cell damage and inflammation [20] and has been described as a promotor of endothelial cell migration and proliferation [21] with anti-inflammatory properties in a setting of endotoxemia [21]. Following MI, CTRP3 was found to exert beneficial effects both on reverse remodeling while promoting mesenchymal cell migration accompanied by anti-oxidative effects [22] and on cardiac fibrosis by inhibition of myofibroblast differentiation [23]. Chen et al*.* reported an improvement in oxLDL-induced inflammation and endothelial dysfunction by CTRP3 [24]. Interestingly, CTRP3 exerted an anti-inflammatory effect by inhibiting cytokine secretion and promoting a switch of circulating monocytes to the intermediate CD14^++^CD16^+^ phenotype after acute MI [25]. There are already some studies investigating CTRP3 in the context of CAD, most of these studies report decreased CTRP3 levels in CAD [18, 26, 27].

In this study, we investigated the plasma levels of patients with chronic coronary syndrome (CCS) compared to patients with ACS after an acute event, and after a recurrent acute event, respectively. We also assessed a possible relationship of CTRP3 levels with age, BMI, and clinical parameters such as circulating inflammatory markers and monocyte subpopulations. Furthermore, CTRP3 levels have been investigated in mouse models of diet-induced atherosclerosis and experimental infarction.

## Material and methods

### Study population

In this single-center study, 206 male and female patients undergoing coronary angiography for the diagnosis and percutaneous intervention of CAD were enrolled in the University Hospital of Giessen and Marburg (UKGM) in the department of cardiology, angiology, and intensive care medicine. Patients received standard cardiovascular care and medication (ACE-inhibitor, AT_1_-receptor blocker, β-blocker, diuretics, statin) according to the current guidelines. We investigated patients with proven coronary artery disease by coronary angiography and stenosis larger than 70% in relevant vessels that require intervention. Patients were classified into 3 groups: 1) chronic coronary syndrome (CCS) patients, in which CAD was diagnosed more than 6 months ago, which in the meantime have had no further signs or symptoms of coronary artery disease under appropriate medication (n = 64), 2) acute coronary syndrome (ACS) patients with first and acute ST-elevation myocardial infarction (STEMI), Non-ST-elevation myocardial infarction (NSTEMI) or unstable angina as diagnosed by electrocardiography. Intervention and diagnosis for the presence of coronary artery was carried out in maximum 4 days before blood sampling was performed (ACS-1, n = 75) and a combination of the two patients groups described before; patients have had the diagnosis of coronary artery disease with intervention 6 months and longer ago and developed a second event (recurrent acute event) of STEMI, NSTEMI or unstable angina with need for intervention in maximum 4 days, before blood samples were collected (ACS-2, n = 67). EDTA blood was collected from each subject, further processing was performed within 2 h. Based on a standardized questionnaire, 49 male and female controls without any history of cardiovascular disease were included in the study. Exclusion criteria were tumor diseases and either missing written informed consent or inability to comply and to understand the investigational nature of the study and participation in other interventional drug or treatment trials. The study itself was conducted in accordance with the guidelines of the Declaration of Helsinki and the research protocol including the case report forms was approved by the local ethics committee (245–12). Written informed consent was obtained from all study participants. The work flowchart for the clinical study is illustrated in Fig. S1A.

### Animal experiments

The animal handling and all experimental procedures were in accordance with the guidelines from directive 2010/63/EU of the European Parliament on the protection of animals used for scientific purposes and were approved by the local animal care and use committee (50/2015, B2/1228).

Experimental atherosclerosis: Low-density lipoprotein receptor-deficient (*Ldlr*¯^/^¯, B6.129S7-*Ldlr*^*tm1Her*^/J) male mice at the age of 10 weeks were either continued on a standard diet (chow diet, CD) or a fed a high-fat diet (HFD, D12079B, containing 21% fat and 0.21% cholesterol, Research Diets, New Brunswick, NJ) for 12 weeks. At the end of this period, blood samples were collected after 4 h of starvation.

Surgical procedures of myocardial infarction: Male C57BL/6 J mice at the age of 10–12 weeks were subjected to permanent or transient ligation of the left anterior descending (LAD). Mice were anesthetized with isoflurane (4%) and intubated using a 20-gauge intravenous catheter with a blunt end. Mice were artificially ventilated with a stroke volume of 1 to 1.5 ml and at a rate of 60–80 strokes per minute using a rodent ventilator with a mixture of O_2_ and air (80%) to which isoflurane (2.0%) was added. The mouse was placed on a heating pad to maintain its body temperature at 37 °C. The chest hair was removed, and the chest was opened in the third intercostal space. Prolene suture (6–0) was used to ligate the left coronary artery. Infarction was confirmed by discoloration of the ventricle and ST-T changes on the electrocardiogram. For transient ligation, the suture was removed after 45 min and reperfusion was allowed. The chest and skin were closed with a 5–0 silk suture. The animals were extubated before they were allowed to recover from the surgery. Sham-operated mice served as controls. Blood was collected after 28 days. The work flowchart for the animal experiments is provided in Fig. S1B.

### Flow cytometry

After washing, PBMCs were stained with anti-human antibodies specific for CD2 (PE, RPA-2.10, T-cell marker), CD14 (APC, M5E2, monocyte subset differentiation), CD15 (PE, HIM1, granulocyte marker), CD16 (PE-Cy7, 3G8, monocyte subset differentiation), CD19 (PE, HIB19, B-cell marker), CD56 (PE, MY31, NK-cell marker), CD335 (PE. 9E2, NK-cell marker), HLA-DR (FITC, TU36, antigen-presenting cells) (all from BD Biosciences, Franklin Lakes, NJ). DAPI was added to identify dead cells. Cells were acquired on a FACS LSR II flow cytometer (BD Biosciences) and analyzed using FlowJo software version 10 (Treestar Inc.).

### Enzyme-linked Immunosorbent Assay (ELISA)

CTRP3 was quantified in plasma samples of cardiovascular patients and controls by applying a human-specific ELISA kit purchased from R&D Systems (Minneapolis, MN, USA; catalog # DY7925-05) and a mouse-specific ELISA kit from Abbexa Ltd (Cambridge, UK) in technical duplicates. Protein concentrations were calculated from optical density by applying a regression standard curve and the coefficient of variation (CV). Measurement was repeated if CV was exceeding 20%.

### Statistical analysis

The software package *SPSS* (IBM, Armonk, NY; version 29.0) was applied in statistical analysis. Pairwise comparisons were made by Mann–Whitney U-test. More than two groups were compared using the Kruskal–Wallis test followed by Dunn’s multiple comparison test. Data are presented as box plots (median with 25th/75th percentile) and whiskers (10th/90th percentile). Spearman’s rank correlation coefficient was used to evaluate associations between variables and ROC curve analysis was performed in order to test for the predictive potential of numerical parameters for classified variables. In general, a *P* value < 0.05 was considered statistically significant.

## Results

### Study population

In total, we enrolled 255 male and female study participants. 206 patients with angiographically documented CAD were included in this study and classified into three clinical cohorts with respect to cardiovascular anamnesis: 1) chronic coronary syndrome (CCS, n = 64), 2) acute coronary syndrome (ACS) having a first acute event (ACS-1, n = 75) and 3) ACS having a recurrent acute event (ACS-2, n = 67). Individuals without any history of cardiovascular disease were included as controls (con, n = 49). Cardiovascular patients were slightly older than controls, had a higher proportion of smokers, had more comorbidities such as arterial hypertension, dyslipidemia, and diabetes mellitus, and were mostly on medication such as antiplatelet treatment, statins, angiotensin-converting enzyme inhibitor/angiotensin II receptor blocker, and β‐blocker. The patients’ basal characteristics are displayed in Table 1. Furthermore, cardiovascular patients had increased circulating levels of the inflammatory marker C-reactive protein (CRP) and ACS patients had additionally increased levels of troponin I (TNI), indicative of myocardial damage. Regarding circulating inflammatory cytokines, we detected higher plasma levels for interleukin (IL)-6. In addition, we analyzed blood monocytes by flow cytometry and found increased levels of total blood monocytes in all cardiovascular cohorts. A more detailed phenotypical classification of monocyte subpopulations based on their cluster of differentiation (CD) 14/16 surface expression revealed subpopulation levels according to a recent study by our group [28] with increased classical (CD14^++^CD16^−^) and intermediate (CD14^++^CD16^+^) monocyte subsets in all patient groups and decreased levels of non-classical monocytes (CD14^+^CD16^++^) in ACS patients. The patients’ inflammatory characteristics are summarized in Table 2.

**Table 1:** Basal patient characteristics

Characteristics	control	CCS	ACS-1	ACS-2
*General*				
n (♂,♀)	49 (24/25)	64 (53/11)	75 (54/21)	67 (55/12)
Age (years)	52.9 ± 6.5	63.4 ± 7.9***	58.1 ± 5.8**^,###^	62.0 ± 7.8***^,§^
Body-mass-index (kg/m^2^)	28.4 ± 5.4	30.6 ± 5.4	28.6 ± 4.7	29.1 ± 5.4
Blood pressure-systolic (mmHg)	135.8 ± 25.2	133.6 ± 19.9	128.4 ± 19.1	135.9 ± 19.2
Blood pressure-diastolic (mmHg)	83.8 ± 13.1	76.5 ± 10.9**	74.8 ± 9.2***	78.4 ± 8.4
Cholesterol				
Total cholesterol (mg/dL)	222.1 ± 36.5	174.3 ± 45.8**	210.2 ± 57.6^##^	173.3 ± 50.5**^,§§^
LDL cholesterol (mg/dL)	135.3 ± 30.1	113.8 ± 34.5	145.6 ± 56.2^##^	111.4 ± 34.4^§§§^
HDL cholesterol (mg/dL)	58.2 ± 16.7	49.1 ± 10.59	51.5 ± 16.6	50.4 ± 17.1
Triglycerides (mg/dL)	159.3 ±	98.4	190.5 ± 136.9	159.1 ± 98.0
*Cardiovascular risk factors, n (%)*				
Smoking (current and former)	25 (51.0)	50 (71.0)**	60 (80.0)**	56 (83.6)***
Arterial hypertension	17 (34.7)	56 (87.5)***	53 (70.7)***^,#^	64 (95.5)***^,§§§^
Dyslipidemia	11 (22.4)	58 (90.6)***	63 (84.0)***	61 (91.0)***
Diabetes mellitus	5 (10.5)	26 (40.6)***	13 (17.3)^##^	22 (32.8)**
Positive family history	14 (28.6)	16 (25.0)	16 (21.3)	27 (40.3)^#^
*Medical treatment, n (%)*				
Antiplatelets	3 (6.1)	50 (78.1)***	54 (72.0)***	64 (95.5)***^,##;§§§^
Statin	4 (8.2)	52 (81.3)***	48 (64.0)***^,#^	62 (92.5)***^,§§§^
ACEI/ARB	8 (16.3)	38 (59.4)***	45 (60.0)***	59 (88.1)***^,#,§§§^
β‐blocker	9 (18.4)	46 (71.9)***	34 (45.3)**^,##^	49 (73.1)**^,§§^

ACS = acute coronary syndrome, CAD = coronary artery disease, CCS = chronic coronary syndrome, ACEI = angiotensin-converting enzyme inhibitor, ARB = angiotensin II receptor blocker, mean ± SD, **P < 0.01, ***P < 0.001 vs. control, ^#^P < 0.05, ^##^P < 0.01, ^###^P < 0.001 vs. CCS,^§^P < 0.05, ^§§^P < 0.01, ^§§§^P < 0.001 vs. ACS-1

**Table 2:** Inflammatory patient characteristics

Characteristics	Control	CCS	ACS-1	ACS-2
*Circulating factors*				
CRP (mg/mL)	5.9 ± 1.4	31.1 ± 7.2**	43.8 ± 9.2***	18.7 ± 4.5*
BNP (ng/mL)	n.r.		435.0 ± 197.5	222.6 ± 108.4
TNI (µg/mL)	n.r.	0.5 ± 0.3	19.3 ± 12.5^##^	3.9 ± 2.1^§§^
IL-1β (pg/mL)	4.0 ± 1.1	3.7 ± 0.9	4.1 ± 1.8	3.6 ± 1.0
IL-6 (pg/mL)	8.0 ± 0.4	10.4 ± 0.9	13.4 ± 1.3**	12.2 ± 1.0*
TNF-α (pg/mL)	10.1 ± 1.2	9.5 ± 1.0	10.9 ± 1.2	9.6 ± 1.3
CCL2 (pg/mL)	67.1 ± 4.3	64.1 ± 8.2	65.2 ± 7.9	61.0 ± 12.5
CTRP3 (ng/mL)	63.6 ± 3.3	42.9 ± 2.4***	47.5 ± 2.3***	43.8 ± 2.5***
*Circulating cells*				
Total leucocytes (× 10^9^)	n.r.	7.7 ± 2.1	9.8 ± 3.5^##^	9.0 ± 3.0
Total monocytes (% PBMCs)	54.4 ± 22.4	65.4 ± 18.0*	63.3 ± 18.4*	65.5 ± 16.6*
Classical (% monocytes)	82.9 ± 19.1	84.1 ± 7.2*	85.2 ± 7.0**	86.7 ± 6.6**
Intermediate (% monocytes)	6.9 ± 2.3	7.1 ± 4.5*	8.3 ± 5.7**	7.5 ± 3.7**
Non-classical (% monocytes)	9.8 ± 4.8	8.9 ± 4.8	6.8 ± 3.8	5.7 ± 3.5*

ACS = acute coronary syndrome, CAD = coronary artery disease, CCS = chronic coronary syndrome, CRP = c-reactive protein, BNP = brain natriuretic peptide, TNI = troponin I, IL-1β = interleukin 1β, IL-6 = interleukin 6, TNF-α = tumor necrosis factor-α, CCL2 = CC-chemokine ligand 2, CTRP3 = C1q/TNF-related protein 3, mean ± SD, *P < 0.05, **P < 0.01, ***P < 0.001 vs. control, ^##^P < 0.01 vs. CCS, ^§§^P < 0.01 vs. ACS-1, n.r. = not recorded

### Circulating CTRP3 levels are decreased in cardiovascular patients

**Fig. 1:**
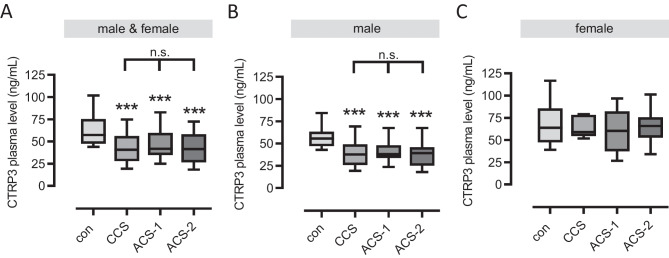
Decreased circulating CTRP3 levels in male cardiovascular patients. Plasma of **A** male and female, **B** only male, and **C** only female cardiovascular patients and controls (con) were analyzed for CTRP3 by ELISA. Patients were subdivided into chronic coronary syndrome (CCS), acute coronary syndrome (ACS), having a first acute event (ACS-1), and ACS having a recurrent event (ACS-2). ***P < 0.001 vs. con. Box plots of the median with 25th/75th percentiles and whiskers with 10th/90th percentiles are shown

**Fig. 2:**
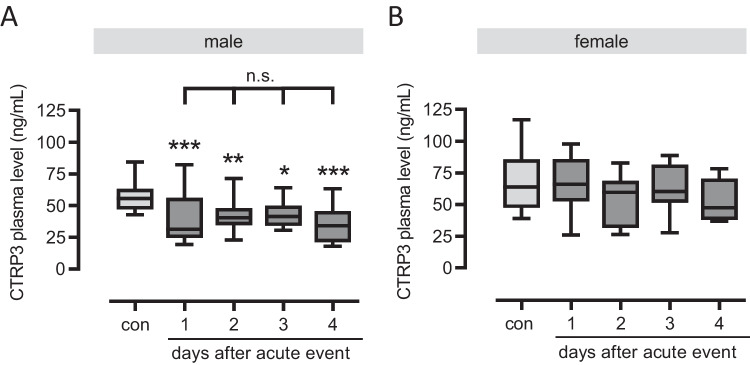
No gradually decreased circulating CTRP3 levels in ACS patients. CTRP3 plasma levels were determined by ELISA of (**A**) male and (**B**) female patients with acute coronary syndrome (ACS) and controls (con). Blood sampling in ACS patients was performed up to day 4 after the acute event. *P < 0.05, **P < 0.01, ***P < 0.001 vs. con. Box plots of the median with 25th/75th percentiles and whiskers with 10th/90th percentiles are shown

Previous studies mostly report decreased CTRP3 levels in CAD patients (Table S1). However, there are also conflicting data and no studies to date comparing chronic and acute patients. Therefore, we included a wide range of cardiovascular patients in our study, chronic (CCS) and acute patients (ACS-1), including those having had a recurrence of an acute event (ACS-2), to determine their CTRP3 plasma levels. Compared to inflammatory cytokines (pg/mL), we detected plasma CTRP3 levels at substantially higher concentrations (ng/mL). From above 60 ng/mL in controls (63.6 ± 3.3 ng/mL), CTRP3 plasma levels decreased significantly to below 50 ng/mL in all of the three cardiovascular cohorts (CCS = 42.9 ± 2.4 ng/mL, ACS-1 = 47.5 ± 2.3 ng/mL and ACS-2 = 43.8 ± 2.5 ng/mL), with no significant difference between CCS, ACS-1, and ACS-2 patients (Table 2 and Fig. 1A). While type 2 diabetes mellitus (T2D) has been reported to be negatively associated with systemic CTRP3 quantities in the literature [29], we detected no significant differences in plasma CTRP3 between diabetic and normoglycemic individuals within all sub-cohorts (data not shown). Decreased CTRP3 quantities in all CAD patient cohorts compared to controls were confirmed for exclusively non-diabetic individuals (data not shown). Interestingly, decreased plasma CTRP3 levels in cardiovascular patients apparently were restricted to males (Fig. 1B) since we did not observe a decrease in female patients (Fig. 1C). However, the female study population was significantly smaller because fewer women than men were included in our study. According to the inclusion criteria, blood sampling in ACS patients was performed up to day 4 after the acute event. In male patients, we observed decreased CTRP3 levels as early as day 1 after the acute event, which then did not decrease significantly further until day 4 (Fig. 2A). Thus, CTRP3 levels decreased rapidly after an acute event rather than slowly over several days. Since female CAD patients did not exhibit decreased CTRP3 levels, we consequently observed no effect here throughout the first four days after blood collection (Fig. 2B). Since average control individuals were slightly younger than the patients within the present study cohort, a correlation analysis of CTRP3 levels with age was performed, without detecting any significant correlation for the entire study cohort (Fig. S2).

### Sexual dimorphism in human circulating CTRP3 levels

**Fig. 3:**
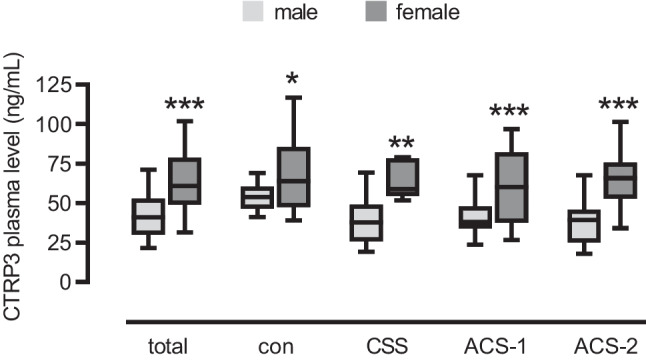
Higher circulating CTRP3 levels in females. **A** CTRP3 plasma levels were determined by ELISA of males and females among the entire study collective (total), controls (con), patients with chronic coronary syndrome (CCS), acute coronary syndrome (ACS) patients having a first acute event (ACS-1), and ACS patients having a recurrent acute event (ACS-2). *P < 0.05, **P < 0.01, ***P < 0.001 vs. male. Box plots of the median with 25th/75th percentiles and whiskers with 10th/90th percentiles are shown

ELISA data analysis revealed that CTRP3 plasma levels were significantly higher in females than in males, by about 20 ng/mL. This was true for the entire collective (+ 21.5 ng/mL) and for all single groups analyzed: control + 14.3 ng/mL, CCS + 24.7 ng/mL, ACS-1 + 18.6 ng/mL, and ACS-2 + 26.1 ng/mL (Fig. 3). Thus, higher CTRP3 circulating levels in women appear to be a general phenomenon and independent of cardiovascular disease. Since CTRP3 did not appear to be regulated in female cardiovascular patients and their proportion in the subgroups was rather low, we focused exclusively on male patients in all further analyses.

### Impact of BMI on circulating CTRP3

In order to investigate a possible interaction of CTRP3 plasma levels with other collected parameters we performed correlation analyses in the male collective. Of note, no significant correlations with anthropometric/ physiological parameters, diagnosed comorbidities, inflammatory cytokines, and monocytes or monocyte subpopulations were observed (Table S2). Among the applied drugs, treatment of CAD patients with diuretics was associated with lower CTRP3 concentrations. Of note, decreased CTRP3 levels in CAD patients when compared to healthy individuals were found to be independent of medication (data not shown).

**Fig. 4:**
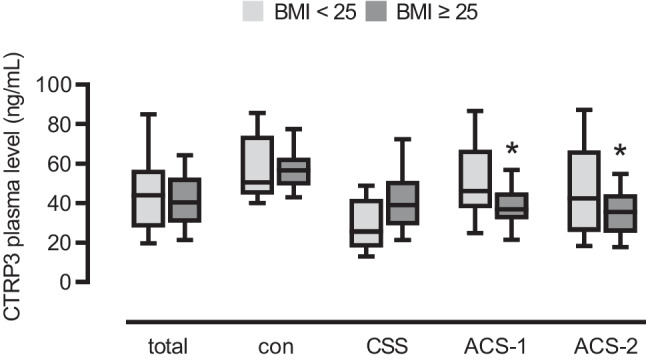
Decreased circulating CTRP3 levels in obese male ACS patients. CTRP3 plasma levels determined by ELISA were categorized by normal-weight (BMI < 25) and overweight (BMI ≥ 25) among the total male study population (total), controls (con), patients with chronic coronary syndrome (CCS), acute coronary syndrome (ACS), with a first acute event (ACS-1), and ACS with a recurrent acute event (ACS-2). *P < 0.05 vs. BMI < 25. Box plots of the median with 25th/75th percentiles and whiskers with 10th/90th percentiles are shown

Since there is a known association between CTRP3 and obesity [11, 30], we divided the male collective of our study into 2 groups, normal-weight subjects (BMI < 25) and overweight subjects (BMI ≥ 25). In the entire collective, in healthy controls, and in CCS patients, there were no significant differences in CTRP3 plasma levels between normal-weight and overweight individuals. However, we found significantly lower CTRP3 plasma levels in overweight ACS-1 and ACS-2 patients when compared to normal-weight patients (Fig. 4).

### Circulating CTRP3 levels as a biomarker for CAD

**Fig. 5:**
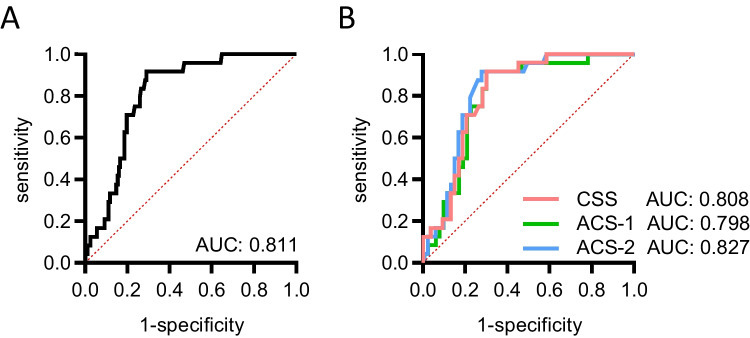
Circulating CTRP3 levels as a predictive marker for CAD. ROC analysis of CTRP3 levels determined by ELISA in males was performed for testing as a potential biomarker for CAD. Comparative ROC analysis was applied for (**A**) the total male study population and separately for (**B**) patients with chronic coronary syndrome (CCS), acute coronary syndrome (ACS), with a first acute event (ACS-1), and ACS with a recurrent acute event (ACS-2). AUC, area under the curve

Based on the observation of decreased CTRP3 plasma levels in chronic and acute CAD patients, ROC curve analysis was performed in order to investigate the predictive potential of systemic CTRP3 levels for CCS and ACS within the present cohort. CTRP3 levels could be identified as an applicable predictor for the general presence of CAD among male study subjects (AUC = 0.811, Fig. 5A). Applying a cutoff value ≤ 51.25 ng/mL provided 70.8% sensitivity (sens) for the detection of individuals with CAD and a 1-specificity (1-spec) value of 19.8% for discrimination of healthy individuals and CAD patients. Of note, CTRP3 was observed to represent an equally valuable predictor of CCS (AUC = 0.808; sens = 70.8%, 1-spec = 20.8% for cut-off 51.05 ng/mL CTRP3), ACS-1 (AUC = 0.798; sens = 70.8%, 1-spec = 20.4% for cut-off 51.25 ng/mL CTRP3), and ACS-2 (AUC = 0.827; sens = 70.8%, 1-spec = 18.2% for cut-off 50.50 ng/mL CTRP3) (Fig. 5B and Tab. S3).

### Circulating CTRP3 levels in murine models of cardiovascular disease

**Fig. 6:**
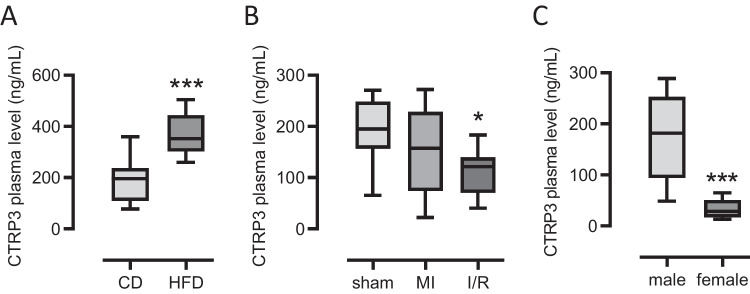
Circulating CTRP3 levels in murine models of cardiovascular disease and gender-specific. **A** Plasma levels of male *Ldlr*¯^/^¯ mice for 12 weeks on a chow diet (CD, n = 10) or a high-fat diet (HFD, n = 11) were analyzed for CTRP3 by ELISA. ***P < 0.001 vs. CD. **B** Plasma levels of male C57BL/6J mice, 4 weeks after either permanent ligation of the left anterior descending artery (myocardial infarction, MI, n = 9) or after transient ligation (ischemia/reperfusion, I/R, n = 7) were analyzed for CTRP3 by ELISA. Sham-operated mice served as controls (n = 7). *P < 0.05 vs. sham. (**C**) Plasma levels from male (n = 22) and female (n = 22) C57BL/6J mice were analyzed for CTRP3 by ELISA. ***P < 0.001 vs. male. Box plots of the median with 25th/75th percentiles and whiskers with 10th/90th percentiles are shown

Finally, we investigated CTRP3 levels in different experimental cardiovascular mouse models. Of note, the measured concentrations for CTRP3 were generally higher in mice than in patients. First, we examined plasma samples from male *Ldlr*¯^/^¯ mice fed a high-fat diet (HFD) for 12 weeks and compared them with samples from *Ldlr*¯^/^¯ mice fed a chow diet (CD) during this period. As was expected, HFD induced experimental atherosclerosis in the *Ldlr*¯^/^¯ mice which was demonstrated by the detection of atherosclerotic plaques in cryosections from the aortic root using Oil Red O staining (Fig. S3). Interestingly, we found higher circulating CTRP3 levels in *Ldlr*¯^/^¯ (365.2 ± 23.8 ng/mL) mice after HFD-induced atherosclerosis than in controls (193.9 ± 27.3 ng/mL, Fig. 6A), which is contrary to our observations regarding the levels in CAD patients (Fig. 1). We next determined circulating CTRP3 levels in mice 28 days after experimental ligation of the left anterior descending (LAD). To this end, we performed both permanent ligation of the LAD (myocardial infarction, MI) and transient ligation (ischemia/reperfusion, I/R) in male wild-type mice (C57BL/6 J). Experimentally induced infarction was confirmed by markedly reduced ejection fraction (EF, Fig. S4) using magnetic resonance imaging. A significant decrease in plasma CTRP3 levels when compared to sham-operated mice (186.4 ± 18.3 ng/mL), according to our findings in ACS patients, was observed exclusively after transient LAD ligation (I/R, 111.2 ± 18.3 ng/mL) but not after permanent ligation (MI, 152.1 ± 28.4 ng/mL) (Fig. 6B). The sexual dimorphism observed in patients was exactly the opposite in mice since females (19.8 ± 6.3 ng/mL) exhibited significantly lower circulating CTRP3 levels than males (179.5 ± 37.6 ng/mL) (Fig. 6C). Thus, substantial differences in circulating CTRP3 levels apparently exist between patients and mouse models in terms of cardiovascular pathophysiology and gender.

## Discussion

CTRP3 – CORS26, cartducin, or cartonectin [31] – was initially characterized as a factor supporting proliferation in various cell types including endothelial and vascular smooth muscle cells [21, 32] and exerting regulatory effects in adipocytes [33]. With its role as a pleiotropic and anti-inflammatory adipokine, it is involved in a plethora of clinical pathologies that have been studied extensively during the past 15 years. For example, CTRP3 has been proposed as a biomarker for nonalcoholic fatty liver disease [34]. However, the vast majority of clinical studies on CTRP3 exist in metabolic and/or cardiovascular contexts. For instance, a negative association with metabolic disorders such as obesity and type 2 diabetes mellitus has been reported [11, 29, 30] and CTRP3 has also been identified as an important player in cardiovascular disease [11].

In the study presented here, we investigated circulating CTRP3 levels in a cohort of patients with different CAD entities and compared them to a control group with angiographically excluded CAD. Similar to several previous studies [18, 26, 27], we report significantly decreased CTRP3 levels in both, CCS and ACS patients. To the best of our knowledge, the present study is the first to comparatively analyze circulating CTRP3 levels in a larger number of ACS patients suffering from acute infarction or re-infarction, respectively. Of note, these two subgroups of our ACS cohort do not exhibit any significant difference regarding circulating CTRP3 levels, also when being compared with the CCS cohort. Thus, circulating CTRP3 appears to be equally responsive to CCS and ACS whilst not further decreasing with recurrent events. The treatment of CAD patients in our study was carried out according to the guidelines with ACE inhibitors, AT1-receptor blockers, β-blockers, diuretics, and statins. Even though the reported divergence of CTRP3 levels in CAD patients and healthy individuals was observed to be independent of treatment with these drugs, we cannot completely rule out an influence of the medication due to the limited size of the study group.

Regarding the whole study cohort, we observe a general sexual dimorphism with higher CTRP3 levels in females than in males. This observation is in good accordance with similar findings reported in the literature [35, 36]. Molecular mechanisms underlying this dimorphism yet remain unclear. A previously reported impact of CTRP3 on Leydig cell testosterone production [37] might indicate a potential direct regulatory interaction of CTRP3 and sex hormone expression. Studies have shown that men develop complications from CAD earlier than women; women only with advanced age, but with worse outcome [38]. This might be due to the fact that women are somewhat protected by estrogen and progesterone until the menopause. Whether higher CTRP3 levels in women are protective in this context is not yet known. Although gestational disorders such as preeclampsia and gestational diabetes might represent general confounders affecting systemic CTRP3 levels, neither of these issues can be answered due to the rather low proportion of women in our study and the general composition of our collective.

Of particular interest, CAD-associated decrease of CTRP3 concentrations occurs exclusively in male patients but not in women. This novel observation is an excellent example of gender differences in biomarker profiles and has important implications for an appropriate prospective study cohort design in future scientific approaches involving CTRP3 in cardiovascular disease. As a consequence, we focused on male patients for further subgroup correlation and ROC analysis. We found no correlations of circulating CTRP3 levels with parameters such as age, BMI, blood pressure, and inflammatory markers. However, overweight ACS patients had significantly lower CTRP3 levels than normal-weight ACS patients, whereas this was not the case in CCS patients. This may reflect the elevated risk of obesity in this sub-cohort.

As a major aim of the present study, we tested for the reliability of CTRP3 as a biomarker for CAD entities applying ROC curve analysis in the study cohort. We identified a cut-off value for CTRP3 of 51.25 ng/ml, below which CAD patients can be distinguished from healthy individuals with considerably high specificity and sensitivity. The observed predictive potency of CTRP3 is independent of overweight and T2D. Within each subgroup, we identified similar CTRP3 cut-off values for the classification of CCS and ACS patients (with first-time or repeated infarction) compared with controls.

Of note, we see a general limitation for the potential use as a biomarker based on the fact that CTRP3 has been reported in a very wide concentration range in previous studies. In our present cohort, we detected CTRP3 plasma levels of 40–70 ng/mL in the individual groups, which is rather in the lower range of published values reported by other studies, ranging from 1 to 900 ng/mL. Even collectives that are comparable in age and sex differ considerably in their plasma CTRP3 levels. This may be due, among other things, to the use of ELISA kits from different manufacturers. However, in some cases, the CTRP3 levels even differ between studies using kits from the same manufacturer. Based on the available data, it is currently not possible to define a universal CTRP3 threshold value for the prediction of cardiovascular disease. Although numerous promising studies exist suggesting CTRP3 as a biomarker for various diseases, assays to measure CTRP3 need to be better standardized in order to gain improved diagnostic or therapeutic statements based on the measured values.

Since CTRP3 represents a pleiotropic adipokine being expressed in multiple organs and tissues, its circulating quantities might be affected by numerous endocrine disorders. In particular, systemic CTRP3 concentrations have repeatedly been reported to be negatively associated with T2D [29]. The proportion of diabetic individuals was higher within the CAD cohorts than among the control group in the present study, therefore T2D might represent a relevant confounder contributing – or at least being related – to decreased CTRP3 quantities. Nonetheless, since no significant divergence of CTRP3 plasma levels in diabetic and normoglycemic individuals was detected within the disease groups, this contribution appears to be limited and not to account for the major differences observed between CAD patients and control subjects. Moreover, CTRP3 does not appear to be specifically regulated in one but in many different diseases [29, 34]. Its utility as a biomarker thus might be more conceivable in a panel together with other biomarkers.

Our results in various mouse models of cardiovascular pathologies indicate that CTRP3 appears to be regulated differently in mice than in human patients at least in part. CTRP3 is increased in atherosclerotic Ldlr-KO mice when compared with non-atherosclerotic Ldlr-KO mice. However, since atherosclerosis was induced by HFD, this observation might also be due to a dietary effect. In the experimental infarct models, the I/R model reflects the situation with reduced CTRP3 levels in ACS patients, whereas the permanent ligation model does not. However, it is important to note that we did not use atherosclerotic animals in these experiments, whereas patients with ACS suffer from atherosclerosis. Also, the observed sex differences are contrary in mice and humans in our study. These differences should be carefully considered for future studies applying mouse models in order to investigate CTRP3 regulation in CVD.

In conclusion, CTRP3 is decreased to a similar extent in male CAD patients and in both, chronic (CCS) and acute patients (ACS). Women generally exhibit higher CTRP3 levels that are not decreased in CCS and ACS. Therefore, CTRP3 might not represent a sufficiently specific and stand-alone biomarker. However, in the context of other biomarkers, the diagnostic utility of decreased circulating CTRP3 levels as a more general inflammatory biomarker in male CAD patients is conceivable.

### Supplementary Information

Below is the link to the electronic supplementary material.

Supplementary file1 (DOCX 14 KB)

Supplementary file2 (PPTX 1.75 KB)

Supplementary file3 (DOCX 17 KB)

Supplementary file4 (DOCX 17 KB)

Supplementary file5 (DOCX 15 KB)

## Data Availability

The datasets generated and analyzed during the current study are available from the corresponding author upon reasonable request.

## References

[1] Dagenais GR, Leong DP, Rangarajan S, Lanas F, Lopez-Jaramillo P, Gupta R, Diaz R, Avezum A, Oliveira GBF, Wielgosz A (2020). Variations in common diseases, hospital admissions, and deaths in middle-aged adults in 21 countries from five continents (PURE): a prospective cohort study. Lancet.

[2] Dwivedi AK, Dubey P, Cistola DP, Reddy SY (2020). Association Between Obesity and Cardiovascular Outcomes: Updated Evidence from Meta-analysis Studies. Curr Cardiol Rep.

[3] Rochlani Y, Pothineni NV, Kovelamudi S, Mehta JL (2017). Metabolic syndrome: Pathophysiology, management, and modulation by natural compounds. Ther Adv Cardiovasc Dis.

[4] Weber C, Noels H (2011). Atherosclerosis: Current pathogenesis and therapeutic options. Nat Med.

[5] Saleh M, Ambrose JA (2018) Understanding myocardial infarction. F1000Res 7:F1000 Faculty Rev-1378. 10.12688/f1000research.15096.1

[6] Galic S, Oakhill JS, Steinberg GR (2010). Adipose tissue as an endocrine organ. Mol Cell Endocrinol.

[7] Luo L, Liu M (2016). Adipose tissue in control of metabolism. J Endocrinol.

[8] Unamuno X, Gómez-Ambrosi J, Rodríguez A, Becerril S, Frühbeck G, Catalán V (2018). Adipokine dysregulation and adipose tissue inflammation in human obesity. Eur J Clin Invest.

[9] Funcke JB, Scherer PE (2019). Beyond adiponectin and leptin: Adipose tissue-derived mediators of inter-organ communication. J Lipid Res.

[10] Nakamura K, Fuster JJ, Walsh K (2014). Adipokines: A link between obesity and cardiovascular disease. J Cardiol.

[11] Guo B, Zhuang T, Xu F, Lin X, Li F, Shan SK, Wu F, Zhong JY, Wang Y, Zheng MH (2020). New Insights Into Implications of CTRP3 in Obesity, Metabolic Dysfunction, and Cardiovascular Diseases: Potential of Therapeutic Interventions. Front Physiol.

[12] Yaribeygi H, Rashidfarrokhi F, Atkin SL, Sahebkar A (2019). C1q/TNF-related protein-3 and glucose homeostasis. Diabetes Metab Syndr Clin Res Rev.

[13] Yang Y, Li Y, Ma Z, Jiang S, Fan C, Hu W, Wang D, Di S, Sun Y, Yi W (2016). A brief glimpse at CTRP3 and CTRP9 in lipid metabolism and cardiovascular protection. Prog Lipid Res.

[14] Kopp A, Bala M, Buechler C, Falk W, Gross P, Neumeier M, Schölmerich J, Schäffler A (2010). C1q/TNF-related protein-3 represents a novel and endogenous lipopolysaccharide antagonist of the adipose tissue. Endocrinology.

[15] Schmid A, Kopp A, Hanses F, Karrasch T, Schäffler A (2014). C1q/TNF-related protein-3 (CTRP-3) attenuates lipopolysaccharide (LPS)-induced systemic inflammation and adipose tissue Erk-1/-2 phosphorylation in mice in vivo. Biochem Biophys Res Commun.

[16] Gao C, Zhao S, Lian K, Mi B, Si R, Tan Z, Fu F, Wang S, Wang R, Ma X (2019). C1q/TNF-related protein 3 (CTRP3) and 9 (CTRP9) concentrations are decreased in patients with heart failure and are associated with increased morbidity and mortality. BMC Cardiovasc Disord.

[17] Yoo HJ, Hwang SY, Hong HC, Choi HY, Yang SJ, Choi DS, Baik SH, Blüher M, Youn BS, Choi KM (2013). Implication of Progranulin and C1q/TNF-Related Protein-3 (CTRP3) on Inflammation and Atherosclerosis in Subjects with or without Metabolic Syndrome. PLoS One.

[18] Choi KM, Hwang SY, Hong HC, Choi HY, Yoo HJ, Youn BS, Baik SH, Seo HS (2014). Implications of C1q/TNF-related protein-3 (CTRP-3) and progranulin in patients with acute coronary syndrome and stable angina pectoris. Cardiovasc Diabetol.

[19] Zhang B, Zhang P, Tan Y, Feng P, Zhang Z, Liang H, Duan W, Jin Z, Wang X, Liu J (2019). C1q-TNF-related protein-3 attenuates pressure overload-induced cardiac hypertrophy by suppressing the p38/CREB pathway and p38-induced ER stress. Cell Death Dis.

[20] Wang F, Zhao L, Shan Y, Li R, Qin G (2019) CTRP3 Protects against high glucose-induced cell injury in human umbilical vein endothelial cells. Anal Cell Pathol (Amst) 2019:7405602. 10.1155/2019/7405602 10.1155/2019/7405602 PMC6681575 31428552

[21] Akiyama H, Furukawa S, Wakisaka S, Maeda T (2007). CTRP3/cartducin promotes proliferation and migration of endothelial cells. Mol Cell Biochem.

[22] Zhang Z, Zhu L, Feng P, Tan Y, Zhang B, Gao E, Wang X, Fan C, Wang X, Yi W (2019). C1q/tumor necrosis factor-related protein-3-engineered mesenchymal stromal cells attenuate cardiac impairment in mice with myocardial infarction. Cell Death Dis.

[23] Wu D, Lei H, Wang JY, Zhang CL, Feng H, Fu FY, Li L, Wu LL (2015). CTRP3 attenuates post-infarct cardiac fibrosis by targeting Smad3 activation and inhibiting myofibroblast differentiation. J Mol Med.

[24] Chen L, Qin L, Liu X, Meng X (2019). CTRP3 Alleviates Ox-LDL–Induced Inflammatory Response and Endothelial Dysfunction in Mouse Aortic Endothelial Cells by Activating the PI3K/Akt/eNOS Pathway. Inflammation.

[25] Zhu H, Ding Y, Zhang Y, Ding X, Zhao J, Ouyang W, Gong J, Zou Y, Liu X, Wu W (2020). CTRP3 induces an intermediate switch of CD14++CD16+ monocyte subset with anti-inflammatory phenotype. Exp Ther Med.

[26] Fadaei R, Moradi N, Baratchian M, Aghajani H, Malek M, Fazaeli AA, Fallah S (2016). Association of C1q/TNF-related protein-3 (CTRP3) and CTRP13 serum levels with coronary artery disease in subjects with and without type 2 diabetes mellitus. PLoS One.

[27] Ahmed SF, Shabayek MI, Abdel Ghany ME, El-Hefnawy MH, El-Mesallamy HO (2018). Role of CTRP3, CTRP9 and MCP-1 for the evaluation of T2DM associated coronary artery disease in Egyptian postmenopausal females. PLoS One.

[28] Witten A, Martens L, Schäfer A-C, Troidl C, Pankuweit S, Vlacil A-K, Oberoi R, Schieffer B, Grote K, Stoll M (2022). Monocyte subpopulation profiling indicates CDK6-derived cell differentiation and identifies subpopulation-specific miRNA expression sets in acute and stable coronary artery disease. Sci Rep.

[29] Moradi N, Najafi M, Sharma T, Fallah S, Koushki M, Peterson JM, Meyre D, Fadaei R (2020). Circulating levels of CTRP3 in patients with type 2 diabetes mellitus compared to controls: A systematic review and meta-analysis. Diabetes Res Clin Pract.

[30] Deng W, Li C, Zhang Y, Zhao J, Yang M, Tian M, Li L, Zheng Y, Chen B, Yang G (2015). Serum C1q/TNF-related protein-3 (CTRP3) levels are decreased in obesity and hypertension and are negatively correlated with parameters of insulin resistance. Diabetol Metab Syndr.

[31] Li Y, Wright GL, Peterson JM (2017). C1q/TNF-related protein 3 (CTRP3) function and regulation. Compr Physiol.

[32] Maeda T, Wakisaka S (2010). CTRP3/cartducin is induced by transforming growth factor-β1 and promotes vascular smooth muscle cell proliferation. Cell Biol Int.

[33] Wölfing B, Buechler C, Weigert J, Neumeier M, Aslanidis C, Schöelmerich J, Schäffler A (2008). Effects of the new C1q/TNF-related protein (CTRP-3) “cartonectin” on the adipocytic secretion of adipokines. Obesity.

[34] Zhou W, Wang Y, Wu Y, Yang J, Xu L, Yang Y (2018). Serum CTRP3 level is inversely associated with nonalcoholic fatty liver disease: A 3-y longitudinal study. Clin Chim Acta.

[35] Wagner RM, Sivagnanam K, Clark WA, Peterson JM (2016). Divergent relationship of circulating CTRP3 levels between obesity and gender: A cross-sectional study. PeerJ.

[36] Schmid A, Gehl J, Thomalla M, Hochberg A, Kreiß A, Patz M, Karrasch T, Schäffler A (2020). Downregulation of ctrp-3 by weight loss in vivo and by bile acids and incretins in adipocytes in vitro. Int J Mol Sci.

[37] Otani M, Kogo M, Furukawa S, Wakisaka S, Maeda T (2012). The adiponectin paralog C1q/TNF-related protein 3 (CTRP3) stimulates testosterone production through the cAMP/PKA signaling pathway. Cytokine.

[38] Davies RE, Rier JD (2018). Gender Disparities in CAD: Women and Ischemic Heart Disease. Curr Atheroscler Rep.

