# Supplementary material for: Decreased circulating CTRP3 levels in acute and chronic cardiovascular patients
Source: J Mol Med (Berl). 2024 Mar 4;102(5):667–77. doi: 10.1007/s00109-024-02426-8 (PMC11055757; doi:10.1007/s00109-024-02426-8)
Supplement: Supplementary file 1 — Supplementary file1 (DOCX 14 KB) [file 109_2024_2426_MOESM1_ESM.docx]

**Supplementary Material and Methods**

**Oil Red O staining**

Mice were killed and the aorta was perfused with PBS after the opening of the hepatic portal vein. The heart, including the aortic root and aortic arch, was dissected after perfusion with PBS. The aortic arch was separated, and snap-frozen in liquid nitrogen, and the heart including the aortic root was embedded in Tissue Tek OCT (Sakura Finetek, Staufen, Germany) for histochemistry. The atherosclerotic burden was demonstrated in serial cryostat sections (8 μm, CM3050S, Leica Microsystems) of the aortic root by Oil Red O (Sigma-Aldrich) staining for 2 h at 60°C.

**Magnetic resonance imaging (MRI)**

Cardiac MRI was performed under volatile isoflurane (1.5% to 2.0%) anesthesia with a Bruker Pharmascan 7.0 T, a custom-built circularly polarized birdcage resonator, and the use of the Early Access Package for Self-gated Cardiac Imaging (Intragate). This type of measurement is based on the gradient echo method (repetition time=44.4 ms; echo time=6.0 ms; field of view=2.20x2.20 cm; slice thickness=1.0 mm; matrix=128x128; repetitions=100). The imaging plane was localized using scout images showing the 4- and 2-chamber view of the heart, followed by acquisition in short axis view, orthogonal on the septum in both scouts. Multiple contiguous short-axis slices consisting of 6 to 8 slices were acquired for complete coverage of the left ventricle. All MRI data were analyzed using Qmass digital imaging software (Medis, Leiden, The Netherlands).
