# Supplementary material for: Decreased circulating CTRP3 levels in acute and chronic cardiovascular patients
Source: J Mol Med (Berl). 2024 Mar 4;102(5):667–77. doi: 10.1007/s00109-024-02426-8 (PMC11055757; doi:10.1007/s00109-024-02426-8)
Supplement: Supplementary file 2 — Supplementary file2 (PPTX 1.75 KB) [file 109_2024_2426_MOESM2_ESM.pptx]

## Slide 1
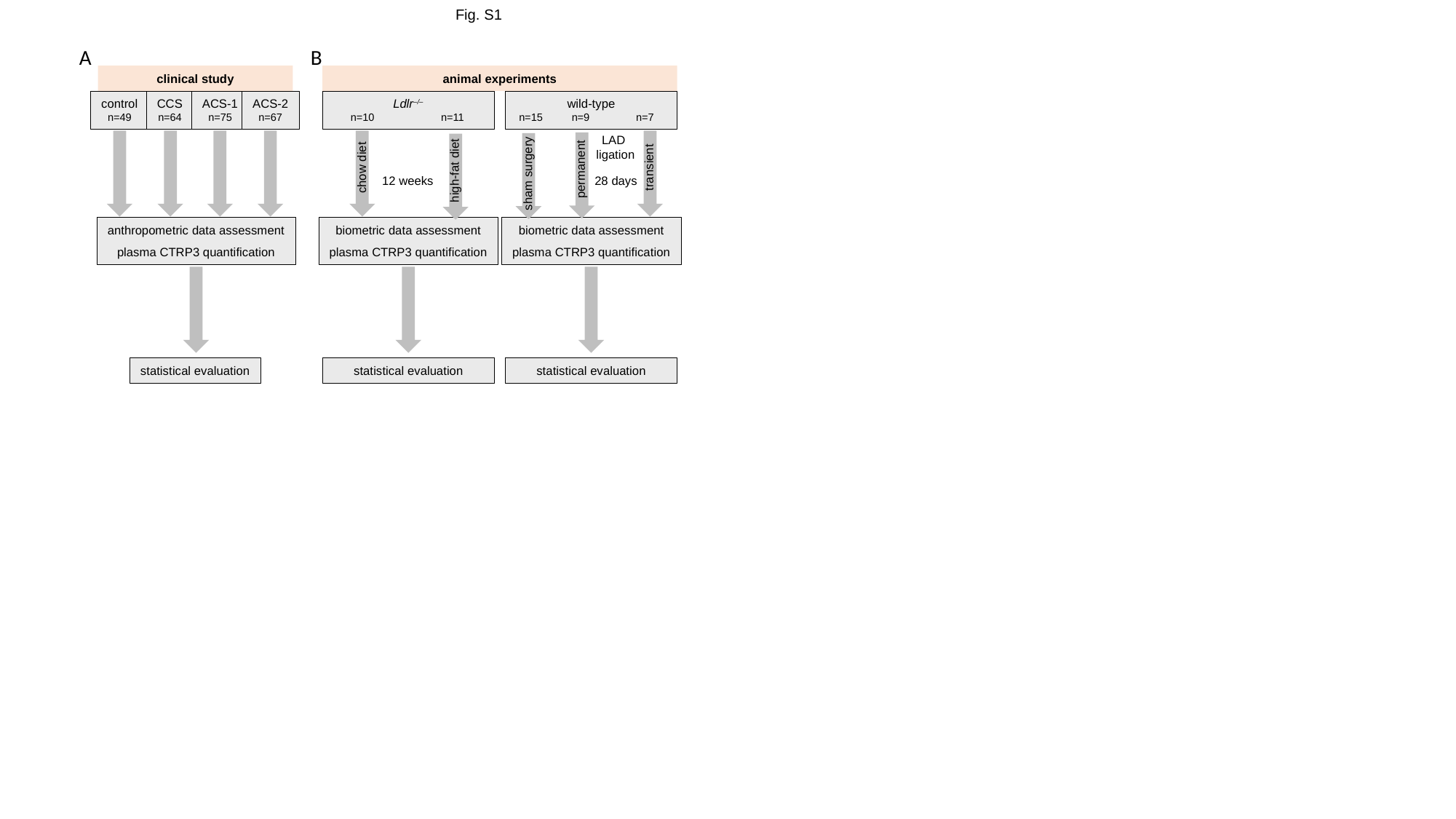

Fig. S1
A
B
clinical study
animal experiments
control
n=49
CCS
n=64
ACS-1
n=75
ACS-2
n=67
Ldlr‒/‒
 n=10 n=11
wild-type
 n=15 n=9 n=7
sham surgery
LAD
ligation
high-fat diet
permanent
chow diet
transient
12 weeks
28 days
anthropometric data assessment
plasma CTRP3 quantification
biometric data assessment
plasma CTRP3 quantification
biometric data assessment
plasma CTRP3 quantification
statistical evaluation
statistical evaluation
statistical evaluation

## Slide 2
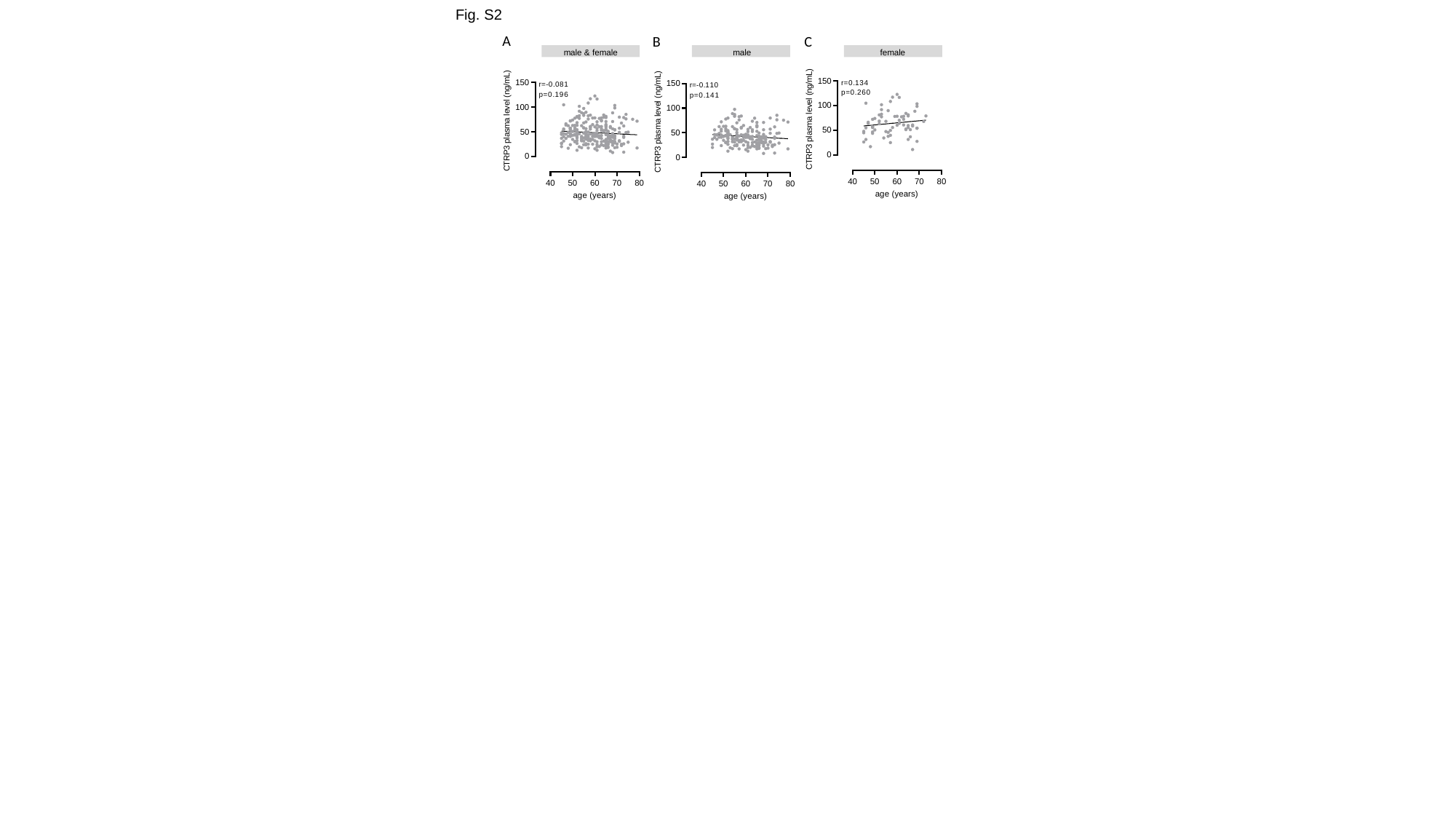

Fig. S2
A
C
B
male
female
male & female

## Slide 3
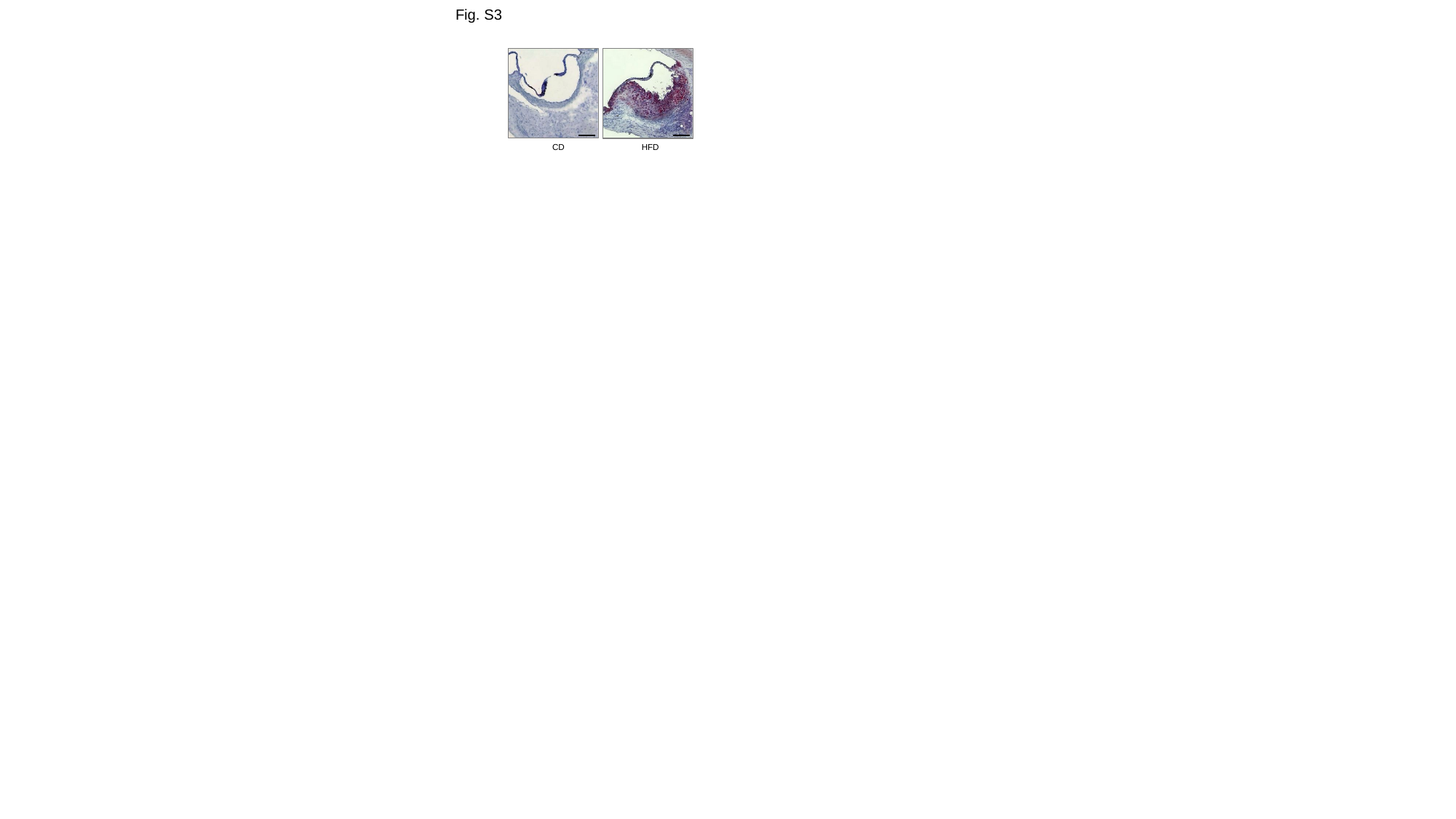

Fig. S3
CD
HFD

## Slide 4
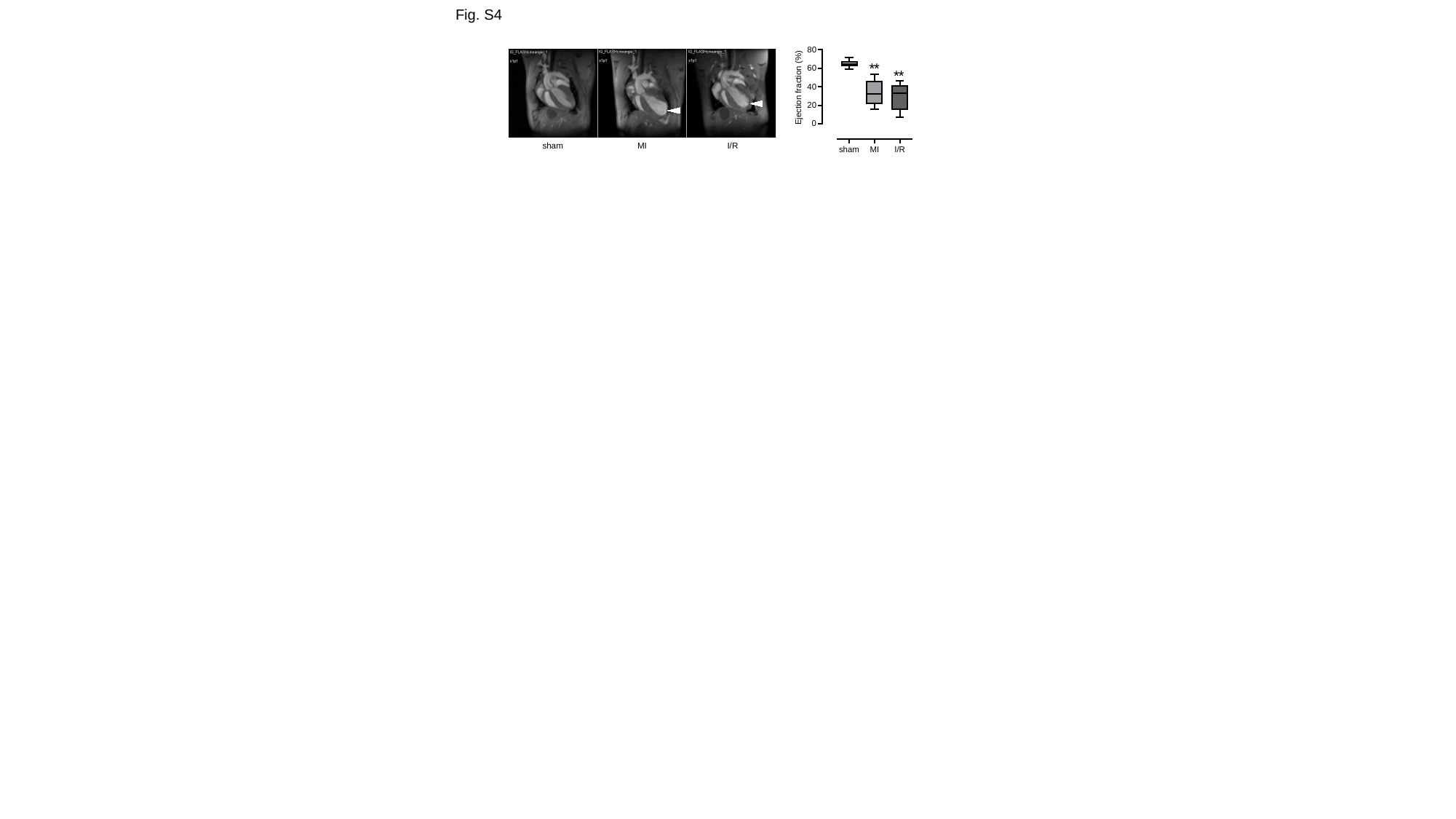

Fig. S4
sham
MI
I/R
