# Supplementary material for: Decreased circulating CTRP3 levels in acute and chronic cardiovascular patients
Source: J Mol Med (Berl). 2024 Mar 4;102(5):667–77. doi: 10.1007/s00109-024-02426-8 (PMC11055757; doi:10.1007/s00109-024-02426-8)
Supplement: Supplementary file 3 — Supplementary file3 (DOCX 17 KB) [file 109_2024_2426_MOESM3_ESM.docx]

**Table S1 – Studies investigating CTRP3 levels in cardiovascular patients.**

year author disease entity group size CTRP3 levels reference (DOI)

(et al.) tendency ng/mL (CTRP3 ELISA manufacturer)

2023 Fan control (no CAD) 21 0.988 10.1016/j.cyto.2022.156119

CAD 27 decreased 1.164 (Meimian Biotech)

2022 Liu control 79 896.0 10.3389/fcvm.2022.967918

ACS 210 696.5 (Jiangsu Meimian Industrial Company)

2022 Matloch control (no CAD) 11 60.2 10.3390/ijms23179988

CAD 14 unchanged 64.8 (R&D Systems)

2022 Yildirim control 50 432.0 10.36660/abc.20200669

CAD 252 decreased 331.0 (Adipogen)

2018 Ahmed control (healthy) 13 130.4 10.1371/journal.pone.0208038

CAD 29 decreased 80.3 (Aviscera biosciences)

2017 Wang control (no CAD) 66 44.1 10.1186/s12872-017-0646-7

CAD 79 increased 56.7 (Senxiong Biotech Industry Company)

2016 Fadaei control (no CAD) 43 305.3 10.1371/journal.pone.0168773

CAD 43 decreased 240.5 (Adipogen)

2016 Wagner control (other diseases) 71 637.8 10.7717/peerj.2573

CAD 29 unchanged 638.4 (Aviscera Bioscience)

2014 Choi control (healthy) 208 410.6 10.1186/1475-2840-13-14

ACS 69 decreased 329.6 (Adipogen)

stable AP 65 decreased 332.9

Summary of a literature search on CTRP3 and cardiovascular patients. The disease entity and controls are as named by the authors of the study. For CTRP3 levels, the mean or median is given. ACS = acute coronary syndrome, AP = angina pectoris, CAD = coronary artery disease.
