# Supplementary material for: Decreased circulating CTRP3 levels in acute and chronic cardiovascular patients
Source: J Mol Med (Berl). 2024 Mar 4;102(5):667–77. doi: 10.1007/s00109-024-02426-8 (PMC11055757; doi:10.1007/s00109-024-02426-8)
Supplement: Supplementary file 4 — Supplementary file4 (DOCX 17 KB) [file 109_2024_2426_MOESM4_ESM.docx]

**Table S2 – Correlation analysis of plasma CTRP3 concentrations with anthropometric, physiological, and inflammatory parameters.**

| parameters correlated with CTRP3 | n | *rho* | *P* |
| --- | --- | --- | --- |
| age | 186 | - 0.110 | 0.141 |
| BMI | 186 | + 0.023 | 0.760 |
| systolic blood pressure | 167 | + 0.099 | 0.202 |
| diastolic blood pressure | 167 | + 0.129 | 0.098 |
| IL-1β | 70 | - 0.035 | 0.776 |
| IL6 | 70 | + 0.007 | 0.957 |
| TNF-α | 70 | + 0.163 | 0.179 |
| CCL2 | 70 | + 0.045 | 0.710 |
| total monocytes | 165 | + 0.019 | 0.807 |
| - classical (CD14^hi^CD16^lo^) | 165 | - 0.044 | 0.571 |
| - intermediate (CD14^hi^CD16^hi^) | 165 | + 0.048 | 0.542 |
| - non-classical (CD14^lo^CD16^hi^) | 165 | + 0.104 | 0.182 |

IL-1β = interleukin 1β, IL-6 = interleukin 6, TNF-α = tumor necrosis factor-α, CCL2 = CC-chemokine ligand 2.
