# Supplementary material for: Decreased circulating CTRP3 levels in acute and chronic cardiovascular patients
Source: J Mol Med (Berl). 2024 Mar 4;102(5):667–77. doi: 10.1007/s00109-024-02426-8 (PMC11055757; doi:10.1007/s00109-024-02426-8)
Supplement: Supplementary file 5 — Supplementary file5 (DOCX 15 KB) [file 109_2024_2426_MOESM5_ESM.docx]

**Table S3 – Predictive quality of CTRP3 plasma levels for cardiovascular morbidities.**

CAD CTRP3 cut-off value specificity sensitivity 1-specificity

CCS+ACS-1+ACS-2 51.25 ng/mL 80.2 % 70.8 % 19.8 %

CSS 51.05 ng/mL 79.2 % 70.8 % 20.8 %

ACS-1 51.25 ng/mL 79.6 % 70.8 % 20.4 %

ACS-2 50.50 ng/mL 81.8 % 70.8 % 18.2 %

ACS = acute coronary syndrome, CAD = coronary artery disease, CCS = chronic coronary syndrome.
